# Pulse Feature-Enhanced Classification of Microalgae and Cyanobacteria Using Polarized Light Scattering and Fluorescence Signals

**DOI:** 10.3390/bios14040160

**Published:** 2024-03-28

**Authors:** Ran Bi, Jianxiong Yang, Chengqi Huang, Xiaoyu Zhang, Ran Liao, Hui Ma

**Affiliations:** 1 School of Physics and Optoelectronic Engineering, Yangtze University, Jingzhou 434023, China; 2021710185@yangtzeu.edu.cn; 2 Shenzhen Key Laboratory of Marine IntelliSense and Computation, Institute for Ocean Engineering, Tsinghua Shenzhen International Graduate School, Tsinghua University, Shenzhen 518055, China; jx-yang21@mails.tsinghua.edu.cn (J.Y.); hcq22@mails.tsinghua.edu.cn (C.H.); 3 Division of Advanced Manufacturing, Tsinghua Shenzhen International Graduate School, Tsinghua University, Shenzhen 518055, China; 4 Hainan Institute, Zhejiang University, Hangzhou 310058, China; zhang_xiaoyu@zju.edu.cn; 5 Guangdong Research Center of Polarization Imaging and Measurement Engineering Technology, Tsinghua Shenzhen International Graduate School, Tsinghua University, Shenzhen 518055, China; mahui@tsinghua.edu.cn

**Keywords:** microalgae and cyanobacteria classification, polarized light scattering, fluorescence, pulse features

## Abstract

Harmful algal blooms (HABs) pose a global threat to the biodiversity and stability of local aquatic ecosystems. Rapid and accurate classification of microalgae and cyanobacteria in water is increasingly desired for monitoring complex water environments. In this paper, we propose a pulse feature-enhanced classification (PFEC) method as a potential solution. Equipped with a rapid measurement prototype that simultaneously detects polarized light scattering and fluorescence signals of individual particles, PFEC allows for the extraction of 38 pulse features to improve the classification accuracy of microalgae, cyanobacteria, and other suspended particulate matter (SPM) to 89.03%. Compared with microscopic observation, PFEC reveals three phyla proportions in aquaculture samples with an average error of less than 14%. In this paper, PFEC is found to be more accurate than the pulse-average classification method, which is interpreted as pulse features carrying more detailed information about particles. The high consistency of the dominant and common species between PFEC and microscopy in all field samples also demonstrates the flexibility and robustness of the former. Moreover, the high Pearson correlation coefficient accounting for 0.958 between the cyanobacterial proportion obtained by PFEC and the cyanobacterial density given by microscopy implies that PFEC serves as a promising early warning tool for cyanobacterial blooms. The results of this work suggest that PFEC holds great potential for the rapid and accurate classification of microalgae and cyanobacteria in aquatic environment monitoring.

## 1. Introduction

As a widely distributed group of microorganisms, microalgae and cyanobacteria create organic compounds through photosynthesis and facilitate the transfer of nutrients through diverse food webs [1,2]. These unicellular organisms play a vital role as the primary producers in aquatic ecosystems, profoundly influencing the abundance of other species [3]. Moreover, high value-added products from microalgae and cyanobacteria have potential applications in health food or medicine, which can bring enormous profits to human society [4]. With the significant impact on ecological dynamics and biogeochemical cycles, it is critical to understand their role in the complex processes of aquatic environments.

However, the proliferation of cyanobacteria in water environments has caused a severe global issue, reported in recent years, due to eutrophication and climate change [5,6,7]. Toxic cyanobacterial blooms lead to depleted oxygen and the release of many cyanotoxins into environments, which poses a serious threat to the biodiversity and stability of local aquatic ecosystems and further to human health [8,9]. As the frequency and duration of blooms continue to increase, it is essential to take measures to prevent and control their dramatic outbreaks [10,11,12]. Hence, a rapid and accurate method that enables the classification and monitoring of harmful algal blooms (HABs) is increasingly desired in water environment management [13].

Monitoring species and concentrations in microalgae and cyanobacteria is significantly complicated by the great variety in sizes, shapes, and pigments [14]. Currently, optical measurement technologies have become a popular tool for observing microalgae and cyanobacteria with the advantages of no contact, real-time measurement, and high resolution [15]. As a common method for cell counting and identification, microscopy requires a high level of expertise and considerable time and effort, which makes it difficult to deploy in the field [13]. Spectral analysis enables rapid measurement of the absorption spectra for biomass and concentration of microalgae and cyanobacteria, usually employing spectrophotometers and hyperspectral remote sensing [16,17]. However, this method is greatly affected by complex environments and fails to classify detailed species. Additionally, flow cytometry utilizes a laser to generate scattering and fluorescence signals from cells, which has been widely applied for microalgae and cyanobacteria classification in the laboratory [18]. Necessary pre-processing such as fluorescence labeling and hydrodynamic focusing should be required, which inevitably limits its field application [19]. Therefore, developing new tools to meet the requirements for rapid and accurate classification remains urgent and challenging.

Polarization, as an inherent property of light, is more sensitive to microscopic structures and carries richer information than non-polarized light [20]. Polarized light scattering has become an emerging optical method employed in various fields, including biological tissue analysis, aerosol detection, and environmental monitoring [21,22,23]. In previous work, the effectiveness and feasibility of using polarized light scattering to characterize the cellular structure of microalgae and cyanobacteria and monitor their status have been achieved and demonstrated [24]. Some typical suspended particulate matter (SPM) has been classified based on in situ data of polarized light scattering in the literature. Furthermore, a particulate detection prototype for rapid and simultaneous measurement of polarized light scattering and fluorescence of individual particles has been developed, which may hold great potential for microalgae and cyanobacteria classification [25].

In this paper, we propose a pulse feature-enhanced classification (PFEC) method, which combines a prototype and strategy for extracting pulse features. The prototype simultaneously measures polarized light scattering and fluorescence signals of individual particles as temporal pulse signals, thereby obtaining pulse features to represent the information of each particle. In the laboratory, the data from microalgae, cyanobacteria, and other SPM formed a database containing 30 species for classification. In the field sample experiments, PFEC revealed the respective proportions of three phyla and the dominant and common species within acceptable tolerances. Moreover, the cyanobacterial proportion given by PFEC was highly correlated with the cyanobacterial density under microscopy. The results shown in this work demonstrate the feasibility and generalization performance of PFEC for the nuanced classification of microalgae and cyanobacteria, which holds great potential in aquatic environment monitoring.

## 2. Materials and Methods

### 2.1. Materials

The cultured samples consist of 19 cultured microalgae species, 2 cultured cyanobacteria species, and 9 common SPM that includes sediments and microplastics (Table 1). These species were cultivated at a stable temperature of 25 °C and under a 12:12 h light–dark cycle with a cool fluorescent light. During the experiment, all samples were sampled from the original specimens and added to a sample pool for measurement. In particular, the samples of microalgae and cyanobacteria phyla used in measurement with the prototype were collected during their stationary phase. During preparations of the dataset, samples were divided into *Chlorophyta*, *Bacillariophyta*, and *Cyanophyta* based on their phyla (purchased from Freshwater Algae Culture Collection at the Institute of Hydrobiology, Wuhan, China; Shanghai Guangyu Biological Technology Co., Ltd., Shanghai, China); other SPM included four types of sediment (purchased from Henan Hanhai Environmental protection Tech Co., Ltd., Zhengzhou, China) and five types of microplastic (purchased from Suzhou Nanomicro Tech Co., Ltd., Suzhou, China).

Field samples were collected from an aquaculture area in Zhuji, Zhejiang, China (120°37′ N, 29°84′ E) on 5 November 2023. During sampling, a noticeable occurrence of HABs was observed in water bodies. A total of eight sites were all sampled at a depth of 0.5 m below the water surface, named CF1, CF2, CF3, CF4, GC1, GC2, GC3, and GC4.

### 2.2. Particulate Detection Prototype

**Figure 1:**
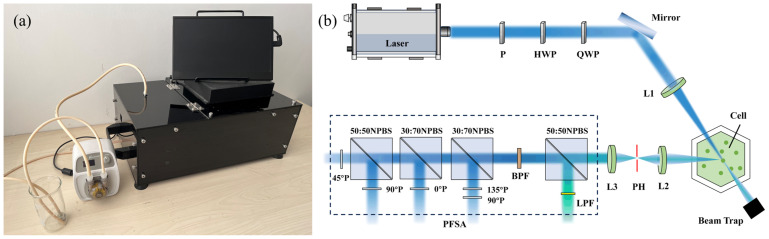
(**a**) Appearance of the prototype; (**b**) schematic illustration of the optical system.

As shown in Figure 1a, the particulate detection prototype involves a main part colored black and a peristaltic pump located externally, which drives sample flow into the optical path for measurement. With the characteristics of integration and portability, it is designed for rapid and accurate classification in laboratory testing and field environmental monitoring.

The optical system of the prototype is illustrated in Figure 1b. The light source is a 1 W laser with a wavelength of 445 nm. The incident light becomes linearly polarized after passing through a polarizer (P) and is modulated to a certain polarization state through a half-wave plate (HWP) and a quarter-wave plate (QWP) in sequence. Subsequently, the light beam is focused on the center of sample pool by a convex lens (L1), where individual particles flow through and can be illuminated. The backscattered light at 120° is collected and focused by a convex lens (L2), while unscattered light is eliminated in the beam trap. Note that the backscattered light passes through a circular pinhole (PH), allowing for only the scattering light from individual particles to pass through. Afterward, a convex lens (L3) collimates the light passing through PH, ultimately entering a polarization and fluorescence state analyzer (PFSA), following our previous work [25]. Once entering the PFSA, the light is divided into five portions by unpolarized beam splitter cubes in a certain proportion. One portion of the light passes through a long-pass filter (LPF) ranging from a wavelength of 460 nm to 950 nm, obtaining fluorescence intensity. The remaining four portions are acquired as four polarization components of the light, which can be combined into a vector of polarization components, that is, [*I*_1_, *I*_2_, *I*_3_, *I*_4_]^T^. It is noteworthy that the scattering volume is limited to 0.01 μL, which ensures that only a single particle at most can be counted in the scattering volume, thereby enabling rapid measurement of individual particles [26].

### 2.3. Data Analysis

Polarized light scattering can obtain high-dimensional polarization parameters. The Stokes vector is usually used to represent the polarization state of light, which consists of four parameters:

(1) $$S=\left[\begin{array}{c} I\\ Q\\ \begin{array}{c} U\\ V \end{array} \end{array}\right]=A\times \left[\begin{array}{c} I_{1}\\ I_{2}\\ \begin{array}{c} I_{3}\\ I_{4} \end{array} \end{array}\right]$$

where *I* represents the total light intensity, *Q* and *U*, respectively, represent the residual polarization at horizontal and 45° polarization directions, and *V* represents the residual circular polarization. A is the instrumental matrix to calibrate the error of the PFSA, which can transfer the measured polarization component vector [*I*_1_, *I*_2_, *I*_3_, *I*_4_]^T^ to the Stokes vector of light.

A can be calculated based on multiple calibration measurements in advance:

(2) $$A=S_{PAX}\times {{(S}_{S})}^{-1}$$

where $S_{S}$ is the Stokes vector of the incident polarized light and $S_{PAX}$ represents the measurement result by a commercial polarization instrument (PAX1000VIS, Thorlabs Inc., Newton, NJ, USA). After adjusting different incident light polarization states for multiple measurements, the value of A can be obtained with a calibration error of less than 3% [25].

As shown in Equation (2), the polarization parameters, that is, *q*, *u*, and *v*, are normalized by the light intensity I:

(3) $$q=\frac{Q}{I},u=\frac{U}{I},v=\frac{V}{I}$$

The degree of polarization (DOP) is commonly used to describe the degree of polarization of light, representing the proportion of polarization parameters in total light intensity. DOP ranges from 0 to 1, where 0 represents non-polarized light, meaning the absence of polarization components in light; 1 represents fully polarized light, indicating that the light is entirely composed of polarization components.

(4) $$DOP=\sqrt{q^{2}+u^{2}+v^{2}}$$

The fluorescence is the intensity of light ranging from a wavelength of 460 nm to 950 nm, denoted as *F*.

### 2.4. Algorithm Theory

#### 2.4.1. Backpropagation Neural Network

The backpropagation neural network (BP-NN) is a common and effective model in artificial neural networks, composed of many interconnected neurons, exhibiting typical features of learning and generalization [27]. A BP-NN consists of an input layer, hidden layer, and output layer, which is characterized by error backpropagation, adjusting connection weights to reduce errors during training.

#### 2.4.2. Quantitative Metrics

The confusion matrix is applied to display actual and predicted categories of the model, which describes the results of algorithm predictions [28]. Each row of the confusion matrix represents the actual category, while each column represents the predicted category. The confusion matrix has four outputs, including true positive (TP), false negative (FN), false positive (FP), and true negative (TN). Additionally, the accuracy can be used to describe the overall performance of the classification model as follows:

(5) $$Accuary=\frac{TP+TN}{TP+FP+TN+FN}$$

The correlation coefficient r represents the degree of association between two variables, that is:

(6) $$r=\frac{Cov(x,y)}{\sqrt{Var(x)Var(y)}}$$

where Cov(x,y) is the covariance of *x* and *y*, Var(x) is the variance of *x*, and Var(y) is the variance of *y*. With values ranging from −1 to 1, the closer r is to 1, the higher the degree of positive linear correlation between two variables; the closer r is to −1, the lower the degree of negative linear correlation between two variables.

### 2.5. Microscopy

The collection and processing of samples mainly follow the methods for freshwater algae research [29]. Eight field samples of 500 mL were collected, and 15% Lugol’s solution was added for fixation. After standing for 24 h, samples were concentrated to 30 mL, shaken horizontally 100–200 times for uniformity. Afterward, 0.1 mL of the sample was taken and placed in a plankton counting chamber. In this work, a microscope (CX-40, Sunny Optical Technology Co., Ltd., Yuyao, China) with a 10 × 40 magnification lens was used for cell counting and identification. The microalgae quantity was determined by counting 50–100 fields of view based on the number of microalgae and cyanobacteria in each field. Note that microscopy in this work is used as a standard tool for identification and cell counting in water that mainly provides the validation of the PFEC method.

### 2.6. Determination of Chlorophyll a

This study utilized a fluorophotometer to measure chlorophyll a (Chl-a). Each water sample, measuring 200 mL, was subjected to filtration treatment. Subsequently, the filter membrane was placed in a grinding device, and 3–4 mL of acetone solution was added before grinding. The liquid extracted from crushed cells was transferred to a cuvette, whose Chl-a content was determined using a fluorescence spectrophotometer (INESA Analytical Instrument Co., Ltd., Shanghai, China).

## 3. Results

### 3.1. Extraction of Pulse Features

**Figure 2:**
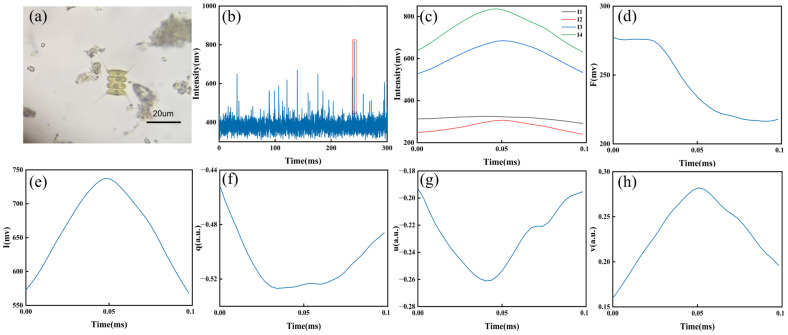
Information on the N8 (*Scenedesmus* sp.) cells: (**a**) optical microscopic image, (**b**) polarization signal in a time series measured by prototype, (**c**) four polarization signals of one cell, and (**d**) fluorescence pulse of one cell. Calculated Stokes vector of one cell, namely, (**e**) *I*, (**f**) *q*, (**g**) *u*, and (**h**) *v*.

To demonstrate the process of extracting pulse features, sample N8 was selected to describe the whole course from signal measurement to feature extraction. As shown in Figure 2a, the outer cells of this sample were a series of fusiform navicular bodies with long curved spines at each pole, and the inner cells were spine-free, similar to those in other research [30]. Their suspensions were measured by prototype to acquire signals in a time series, where the high pulse marked as a red rectangle represented one single cell (Figure 2b). Its measured polarization signals (*I*_1_, *I*_2_, *I*_3_, and *I*_4_) were collected, where the width represented the staying time of one particle in the scattering volume, and the value described the intensity of various polarization components (Figure 2c). Note that these signals generally presented synchronous but imperfect shapes.

Subsequently, the fluorescence pulse obtained from same cell was averaged as one value *F* to represent the fluorescence information (Figure 2d). Meanwhile, the Stokes vector, *I*, *q*, *u*, and *v*, could be calculated (Figure 2e–h). The profile of *I* was usually similar to the signal of *I*_1_. Additionally, the other pulses normalized by *I* were different from their corresponding signals, which might be subjected to both the energy density distribution of illuminating light and the route that the single cell passed through the scattering volume. Polarization pulses contained more high-dimensional details on the backscattered light than the intensity pulse *I*, demonstrating great potential for classification. In previous studies, four polarization pulses were usually represented by the pulse-average classification (PAC) method [25], ignoring their pulse features, which might contain a wealth of particle information.

In this work, eight pulse features (as shown in Table 2) were applied to extract pulse features of each polarization pulse signal for enhancing classification. For instance, $X_{Peak}$, $X_{var}$, $X_{std}$, and $X_{rms}$ are related to the distributions of one pulse, which may reveal the intuitive physical meanings of the pulse signal [31]. $X_{sk}$ is a descriptive statistic to describe the skewness and degree of pulse distribution, and $X_{ku}$ characterizes the shape of the pulse distribution relative to a normal distribution, measuring its sharpness or flatness [32]. S is a statistic of the RMS value to the average value and commonly describes the flatness of signal, which may be related to specific waveforms [33]. L is a statistic of the peak-to-peak value to the RMS value, providing information about the signal peak and describing the sharpness of one waveform [34].

### 3.2. Classification Model for Microalgae, Cyanobacteria, and Other SPM

**Figure 3:**
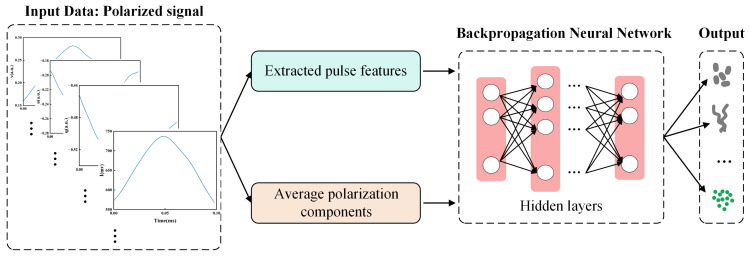
Flow chart of classifier based on BP-NN model.

Herein, a database of four categories containing microalgae, cyanobacteria, and other SPM in Table 1 was established. Each category measured 48,000 data points, for a total database size of 192,000. Note that each data point provided 38 pulse features for describing corresponding signals of one particle, including the average polarization components ($\bar{I}$, $\bar{q}$, $\bar{u}$, $\bar{v}$, DOP, and *F*) and 32 extracted pulse features of four polarization components. The data were input into a classifier based on the BP-NN model, which was divided into training, validation, and test sets with a ratio of 60%:20%:20%. The model was set up with five hidden layers, with the numbers of neurons being 64, 128, 256, 128, and 64 (Figure 3).

**Figure 4:**
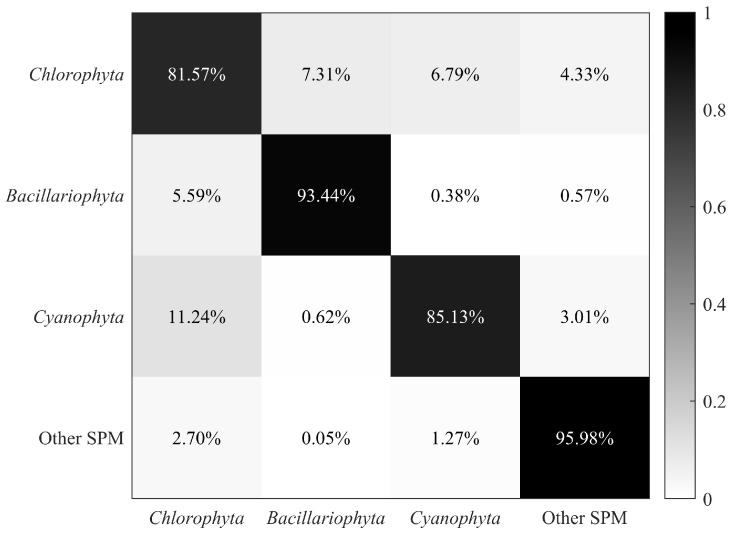
Confusion matrix of microalgae, cyanobacteria, and other SPM.

The classification results of microalgae, cyanobacteria, and other SPM are presented as a confusion matrix shown in Figure 4. The classification model had an overall accuracy of 89.03%, with each accuracy larger than 80%. Particularly, *Bacillariophyta* and other SPM were best classified, with an accuracy larger than 90%, while *Chlorophyta* had the minimum classification accuracy, which was close to 82%. There remained some misidentifications in the *Chlorophyta* and *Cyanophyta* results, which might have been caused by the limited size of the database and the complexity of the classification model. Considering that the three phyla in Table 1 cover multiple microalgae and cyanobacteria species, the results indicate that the model exhibits convincing performance in classification.

### 3.3. Comparison of Classification Results between PFEC and Microscopy

In the field experiments, eight field samples in the aquaculture area were collected and analyzed using PFEC. Each water sample was measured using the prototype, conducting one cycle exactly to avoid repeated sampling. The classification model trained in the last subsection was applied to obtain proportions of microalgae, cyanobacteria, and other SPM in each sample (Table 3). One could see that proportions of other SPM at each site fluctuated little and held stable at a baseline of 50%, indicating that the turbidity levels between different sites were close. Similarly, the *Bacillariophyta* phyla proportion stayed the lowest at all sites, around 8%, which revealed that diatom cells were at a competitive disadvantage in this environment. The proportions of microalgae and cyanobacteria could be measured with the rapid and accurate measurement of PFEC, which may facilitate efficient monitoring of HABs in aquaculture.

**Figure 5:**
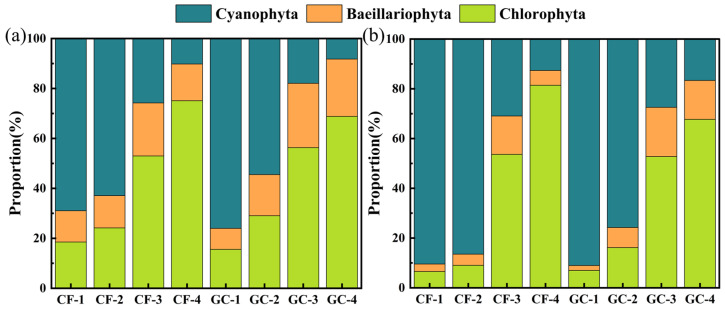
Proportions of three phyla at each site with (**a**) classification results of PFEC and (**b**) classification results of microscopic observation.

To verify the PFEC results of field samples, microscope observation was also applied to identify and count microalgae and cyanobacteria proportions from the same field sample. Meanwhile, we removed the other SPM and adjusted the relative proportions of *Cyanophyta*, *Bacillariophyta,* and *Chlorophyta* to facilitate comparison with the microscopy results as shown in Figure 5a. CF-1, CF-2, GC-1, and GC-2 sites were dominated by *Cyanophyta*, while the other sites were dominated by *Chlorophyta*. For all sites, the proportions of *Bacillariophyta* were less than 25%. Figure 5b shows the proportions of three phyla by microscopy to validate the PFEC method. It was found the sites dominated by *Cyanophyta* and *Chlorophyta* were the same as in PFEC. Similarly, the proportion of *Bacillariophyta* was relatively minor, but lower than for the PFEC results.

There is no doubt that the dominant phylum in field samples can be correctly indicated by PFEC. The proportion of cyanobacterial cells in aquatic environments has been of great concern due to their release of harmful cytotoxins to aquaculture [8,9,35]. Compared with microscopic observation results, the PFEC method showed an average error of 13.36% in *Cyanophyta* proportion, with a maximum difference of 23.56% at site CF-2 and a minimum difference of 2.41% at site CF-4. Considering the time-consuming nature of microscopic observation, PFEC can accurately classify the dominant phylum with acceptable errors. Comparison between PFEC and microscopy demonstrates that PFEC is feasible to provide a potential alternative for the rapid monitoring of natural water bodies.

## 4. Discussion

### 4.1. Comparison of Classification Results between the PAC and PFEC Methods

**Figure 6:**
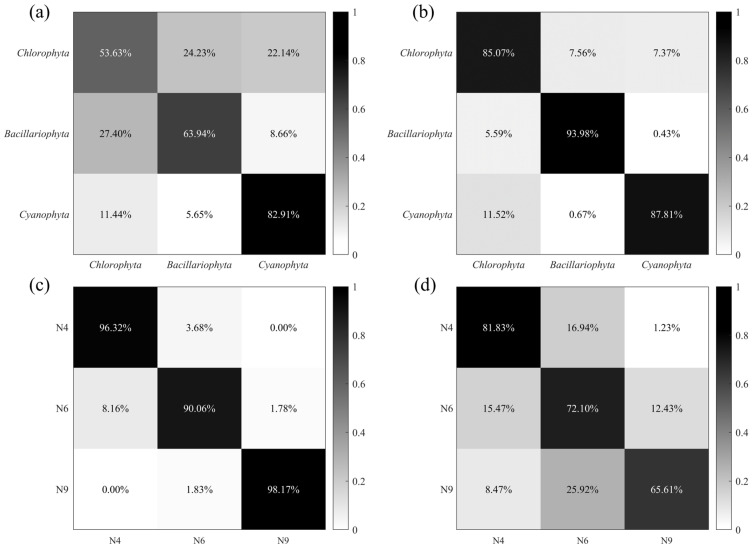
Confusion matrixes of three phyla with two methods: (**a**) PAC ($\bar{I}$, $\bar{q}$, $\bar{u}$, $\bar{v}$, DOP, and *F*) and (**b**) PFEC (38 extracted pulse features). Confusion matrixes of N4 (*Cosmarium* sp.), N6 (*Crucigenia* sp.), and N9 (*Staurastrum* sp.) species with two methods: (**c**) PAC and (**d**) PFEC.

To analyze the contribution of different pulse features to microalgae and cyanobacteria classification, both methods, PAC ($\bar{I}$, $\bar{q}$, $\bar{u}$, $\bar{v}$, DOP, and *F*) and PFEC (38 extracted pulse features), were input into the BP-NN for training, with the dataset of three phyla. The training result of PAC as a confusion matrix is shown in Figure 6a. Although the accuracy of *Cyanophyta* was the highest at 82.91%, the overall accuracy was only 66.59%, since the classification accuracy of the other two phyla was below 65%. Comparing PFEC results in Figure 6b, it could be found that the classification accuracy of each category of microalgae and cyanobacteria was greatly improved, with the maximum increase in accuracy for *Chlorophyta* being 31.44%. Furthermore, the overall classification accuracy increased by 22.36% to 88.95%.

Herein, three *Chlorophyta* species with significant morphological differences, N4 (*Cosmarium* sp.), N6 (*Crucigenia* sp.), and N9 (*Staurastrum* sp.), were also selected to further demonstrate the enhancement of *Chlorophyta* using the extraction of pulse features. In the PAC result, N9 had the lowest classification accuracy of 65.61%, with an overall classification accuracy of 73.18% (Figure 6c). Meanwhile, with the extraction of pulse features, the classification model significantly improved the accuracy of classifying all three species (Figure 6d). The overall classification accuracy reached 94.85%, an improvement of 29.24% over the former. Notably, the accuracy of N6 increased by approximately 32.56%. The extraction of pulse features can provide more detailed information on a single cell. Hence, PFEC can improve the performance and accuracy of the classification model without expanding the size of the database.

### 4.2. Origin of Pulse Feature-Enhanced Classification of Microalgae and Cyanobacteria

**Figure 7:**
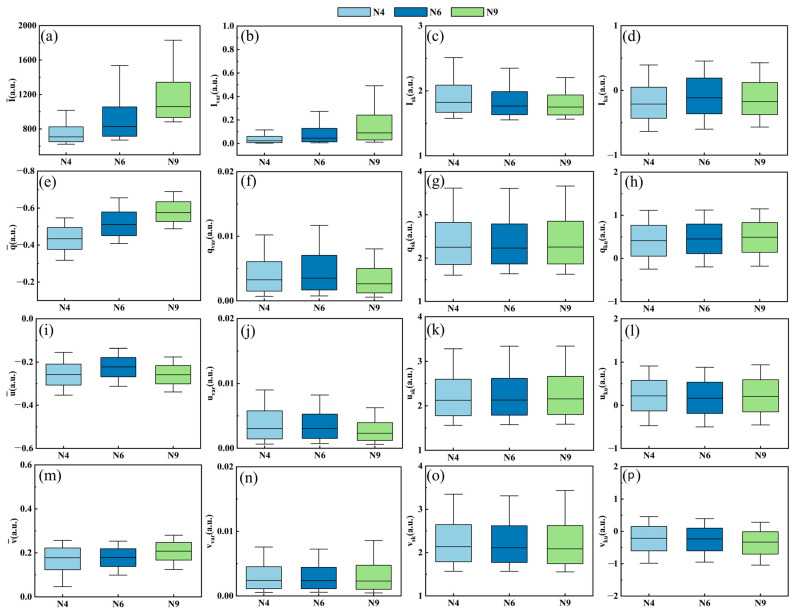
Comparison of N4, N6, and N9 pulse features: (**a**) $\bar{I}$, (**b**) $I_{var}$, (**c**) $I_{sk}$, (**d**) $I_{ku}$, (**e**) $\bar{q}$, (**f**) $q_{var}$, (**g**) $q_{sk}$, (**h**) $q_{ku}$, (**i**) $\bar{u}$, (**j**) $u_{var}$, (**k**) $u_{sk}$, (**l**) $u_{ku}$, (**m**) $\bar{v}$, (**n**) $v_{var}$, (**o**) $v_{sk}$, and (**p**) $v_{ku}$.

To reveal the enhancing effect of PFEC on microalgae and cyanobacteria classification, average values and partly extracted pulse features are shown in Figure 7. The rows of subplots are the pulse features of corresponding polarization parameters (*I*, *q*, *u*, and *v*) and the columns of the subplots are $\bar{X}$, $X_{var}$, $X_{sk}$, and $X_{ku}$, which, respectively, represent the first, second, third, and fourth standardized pulse feature moments to describe the escalating order details of one pulse signal. The first column displays the average values, $\bar{I}$, $\bar{q}$, $\bar{u}$, and $\bar{v}$, of the N4, N6, and N9 species. Note that there were significant differences in $\bar{I}$ and $\bar{q}$ among these species, while there were small differences in $\bar{u}$ and $\bar{v}$, meaning that the latter two parameters contributed little to classification. As for the first row, a main discrimination among the three species was presented in the average values $\bar{I}$ and the variance $I_{var}$, which were the first and second standardized statistics, respectively. The skewness $I_{sk}\;$ seemed to be less discriminating than the former two, and the kurtosis $I_{ku}$ showed little difference. Similar trends could be found in the rows of polarization parameters *q*, *u*, and *v*. Among the different degrees of values, the first and second standardized moment of polarization parameter *I* demonstrated the highest discrimination, which may characterize the different physical properties in the microalgal and cyanobacterial cells.

**Figure 8:**
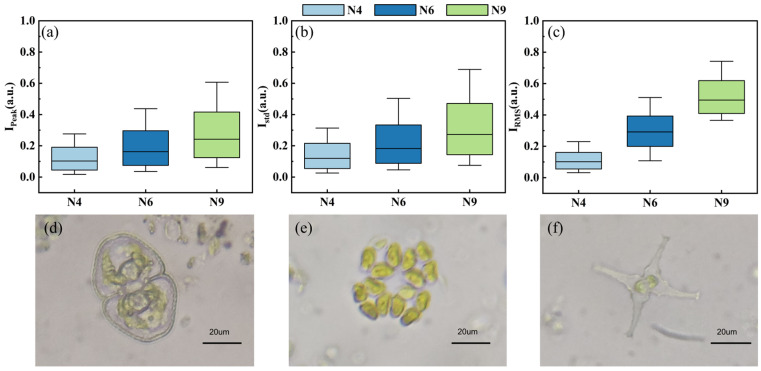
Statistical values in the first degree: (**a**) $I_{peak}$, (**b**) $I_{std}$, and (**c**) $I_{RMS}$; optical microscope images of (**d**) N4, (**e**) N6, and (**f**) N9.

Figure 8a–c shows other statistical values in the first degree including $I_{peak}$, $I_{std}$, and $I_{RMS}$, which may characterize different aspects of the physical properties of microalgae and cyanobacteria. It was found that the selected pulse features exhibited significant differences among the three species, with the highest dispersion and fluctuation in N9, which could explain why N9 achieved the highest classification accuracy. Meanwhile, the optical microscope images of the three species are shown in Figure 8d–f. The N4 cell exhibited a relative peanut shape with a clear membrane and intracellular structures (Figure 8d). The N6 cell was arranged in an elliptical shape with four cells and displayed a cross-shaped gap in the middle (Figure 8e). The difference in the N9 cell was that it had a star-shaped cell body with two smoothly elevated and bifurcated spikes at sides, presenting the most irregular morphological characteristics among the three species (Figure 8f). The various structures of microalgae and cyanobacteria allow for their polarized light scattering and specific fluorescence signals.

The distribution of these extracted pulse features indicates the intrinsic relationship between pulse features and their morphology. In the pulse features among these microalgae and cyanobacteria species, a more irregular morphology usually leads to more dispersed values in the first degree such as in $\bar{X}$, $X_{peak}$, $X_{std}$, and $X_{RMS}$ and has less influence on distributions of a higher-degree value. Even though higher standardized moments demonstrate lower discrimination, it should be noted that the extracted pulse features describe more detailed information on pulse signals than only average values, which improves the model performance and holds the potential for rapid and accurate classification among different microalgae and cyanobacteria.

Recalling the working principle of the prototype, as one particle passes through the scattering volume, its transient route may randomly change, leading to variations in its scattered polarization states and signal profiles. Therefore, pulse features encapsulate many details in single cells, including their morphological characteristics, which thereby results in enhanced classification ability.

### 4.3. Classification of the Dominant and Common Species Using PFEC

In this work, dominant species are considered as those whose proportion exceeds 10% of total microalgae and cyanobacteria, and common species are those within a range of 1% to 10%. Using microscopy, the dominant and common species at different sites were collected (Table 4). It was found that the N20 species dominated microalgae and cyanobacteria at each site, whose proportions were larger than 10%. Additionally, N8 was the dominant species at sites CF-3, CF-4, GC-3, and GC-4, while N9 was the dominant species at sites CF-3, GC-3, and GC-4. As for N10 and N12, they only occurred as the common species at a few sites.

**Figure 9:**
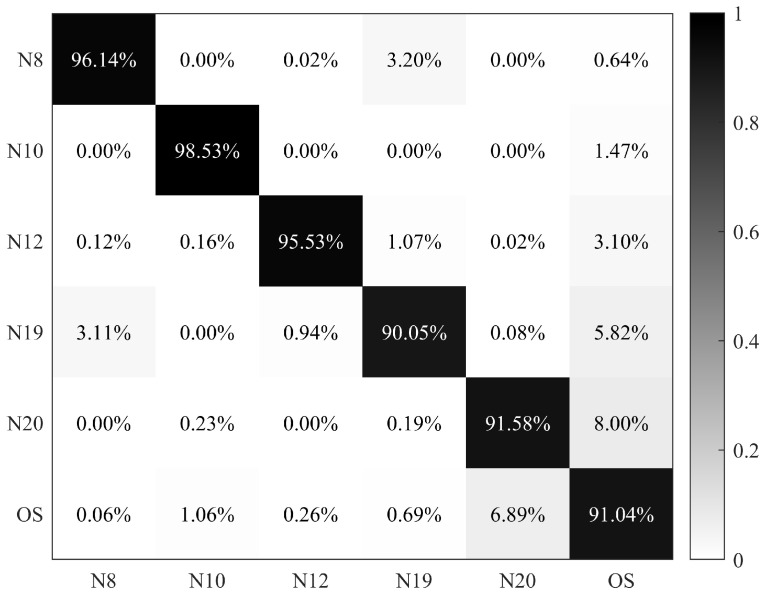
Confusion matrix of N8 (*Scenedesmus* sp.), N10 (*Lagerheimia ciliata*), N12 (*Selenastrum capricornutum*), N19 (*Cyclotella meneghiniana*), N20 (*Microcystis* sp.), and other species (OS).

Herein, based on the dominant and common species given by microscopy, a nuanced classification model based on a BP-NN was built specifically the dominant and common species at these sites. In this dataset, microalgae and cyanobacteria species, including N8, N10, N12, N19, and N20, were treated as separate categories, and other species (OS) in Table 1 were considered as a single category. Classification results were presented as a confusion matrix, with the accuracy for each category above 90% and an overall accuracy of 93.81% (Figure 9).

Applying the nuanced classification model in field samples, the dominant and common species could also be found by PFEC (Table 5). Compared with the microscopic results in Table 4, different estimations were marked with stars. It was found that the discrimination of N20 was the same as the dominant species for all sites except GC-4. However, close observation showed that the proportions of N20 in GC-4 were also close to 10%. Notably, errors occurred in the N8 and N19 classification of four water samples. Similarly, their proportions were both close to 10%. Despite some different estimations, it is clear that there is still more than 78% accuracy of estimation in Table 5. If taking no account of the cases close to 10%, the dominant species can be accurately classified using this nuanced classification model.

PFEC ensures the success of the nuanced classification model in determining the dominant and common species at different sites of natural water bodies. It is encouraging that the flexibility of PFEC enables a customized classification model to specifically identify certain microalgae and cyanobacteria species.

It should be noted that microscopic observation provides clues about the dominant and common species. PFEC independently gives similar results based on that scope, demonstrating its great potential for rapid and accurate monitoring of the water environment. However, it remains challenging to classify all species in Table 1. Similar physical properties of some species in the same phylum may lead to major difficulties in classification, as reported by other optical measurements [14,36,37,38]. By increasing the amounts of data on individual particles and applying improved models, it is anticipated that PFEC has the potential to classify all species listed in Table 1 in further studies.

### 4.4. Correlation Analysis of Cyanobacterial Proportion and Chl-a

**Figure 10:**
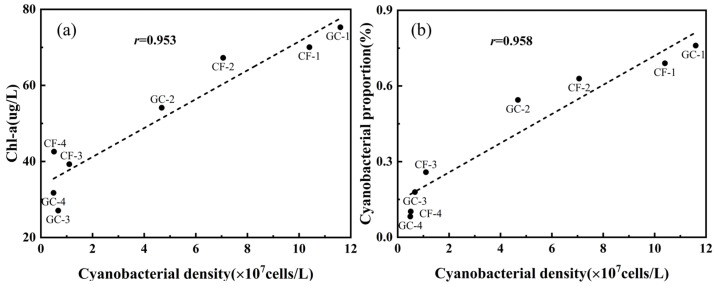
(**a**) Correlation between cyanobacterial density and Chl-a concentration. (**b**) Correlation between cyanobacterial density and cyanobacterial proportion given by PFEC.

Cyanobacterial density given by microscopy is a common direct indicator for monitoring cyanobacterial blooms but requires time-consuming observation [39]. Chl-a concentration has a close relationship with cyanobacterial density, which has so far been used as the main recommended metric to define the level of cyanobacterial blooms for rapid measurement in the environment [40,41]. In our field experiments, Chl-a concentrations of field samples were obtained using a fluorophotometer, which showed a strong correlation with cyanobacterial density with the *r* value of 0.953 (Figure 10a). At sites CF-3, CF-4, GC-3, and GC-4, the cyanobacteria species labeled N20 were dominant in water, so the large cyanobacterial density led to high Chl-a concentrations. However, for the other sites, relatively low cyanobacterial density showed a complex relationship. For example, in Figure 10a, at the sites with low cyanobacterial density, Chl-a concentration fluctuated in the dynamic range from 27 to 43 μg/L. Therefore, the correlation relationship between cyanobacterial density and Chl-a concentration seemed to be significantly higher than other values in the previous literature, but this was understandable [42,43,44].

Section 3.3 demonstrated the ability of PFEC to rapidly and accurately classify various cells in samples, and then cyanobacterial proportion could also be easily estimated. A close positive correlation was also found between cyanobacterial density and cyanobacterial proportion (Figure 10b), whose *r* reached 0.958, close to the Chl-a concentration. It could be found that cyanobacterial proportion increased monotonically with cyanobacterial density. Due to the rapid and accurate classification of microalgae and cyanobacteria using PFEC, the cyanobacterial density could also be obtained easily via evaluating its proportion in the field. However, due to the complexity of aquatic ecosystems, microalga and cyanobacteria always interact and even compete with each other, whose relationship is subjected to various physical and chemical conditions. As such, retrieving cyanobacterial density at a large range by PFEC is still uncertain and inconclusive, which requires further work to confirm its reliability under different conditions and to understand the intrinsic mechanism.

Note that these correlations are based on current field samples, which lack extensive data distributed over time and space. Previous studies have shown this relationship varies with season and environment, resulting in low correlations and potential limitations [45]. Due to the high linear correlation at large cyanobacterial densities, a new approach that combines Chl-a concentration and cyanobacterial proportion may provide a promising tool for rapid monitoring of cyanobacterial blooms. Therefore, we will conduct further research to establish the correlation relationship and demonstrate the reliability of PFEC in obtaining cyanobacterial information in the field.

## 5. Conclusions

This paper proposes a pulse feature-enhanced classification (PFEC) method for rapid and accurate microalgae and cyanobacteria classification in water. The developed prototype can rapidly and simultaneously acquire the polarized light scattering and fluorescence signals from individual particles as a series of temporal pulses. Pulse features of the scattered polarization signals can be extracted by the PFEC proposed and enhance classification performance. The classification model, based on a BP-NN, can effectively classify the dataset consisting of microalgae, cyanobacteria, and other SPM, with an accuracy of 89.03%. Compared with microscopic observation, PFEC could effectively classify three phyla in field samples. In contrast, the classification accuracy of three phyla without pulse features decreased to 66.59%, indicating the importance of the pulse features in accurate classification. Moreover, the comparison between the microscopic images and distribution of pulse features revealed the close relationship between the physical properties of individual cells and the mathematical meanings of pulse features. The flexibility and robustness of PFEC were also demonstrated by classifying the dominant and common species in natural water, which was consistent with the microscopy results. The cyanobacterial proportion predicted by PFEC showed a strong correlation coefficient with cyanobacterial density in the field. In summary, this work demonstrates the power of PFEC in the rapid and accurate classification of microalgae and cyanobacteria in water, which ensures its great potential as a promising tool in future environment monitoring.

## Figures and Tables

**Table 1:** Detailed information on the cultured samples.

Category	Variety	Abbreviation
*Chlorophyta*	*Pandorina* sp.	N1
	*Eudorina* sp.	N2
	*Closterium* sp.	N3
	*Cosmarium* sp.	N4
	*Kirchneriella* sp.	N5
	*Crucigenia* sp.	N6
	*Tetraedron* sp.	N7
	*Scenedesmus* sp.	N8
	*Staursatrum* sp.	N9
	*Lagerheimia ciliata*	N10
	*Chlorella* sp.	N11
	*Selenastrum capricornutum*	N12
*Bacillariophyta*	*Synedra ulna*	N13
	*Asterionella Formosa*	N14
	*Fragilaria nanana*	N15
	*Melosira* sp.	N16
	*Navicula* sp.	N17
	*Nitzschia* sp.	N18
	*Cyclotella meneghiniana*	N19
*Cyanophyta*	*Microcystis* sp.	N20
	*Chroococcus* sp.	N21
Other SPM	Silica sand (18 μm)	N22
	Silica sand (53 μm)	N23
	Quartz powder (38 μm)	N24
	Quartz powder (75 μm)	N25
	Monodisperse polystyrene microspheres (0.5 μm)	N26
	Monodisperse polystyrene microspheres (2 μm)	N27
	Monodisperse polystyrene microspheres (5 μm)	N28
	Monodisperse polystyrene microspheres (8 μm)	N29
	Monodisperse polystyrene microspheres (10 μm)	N30

**Table 2:** Extraction of pulse features (*μ* represents the average).

Pulse Feature	Formula	Physical Property
$X_{Peak}$	max(X)−min(X)	Peak-to-peak value of the signal
$X_{var}$	$\frac{1}{N}\sum_{i=1}^{N}{\left(X_{i}-\mu \right)}^{2}$	Variance (the second standardized moment)
$X_{std}$	$\sqrt{\frac{1}{N}\sum_{i=1}^{N}{\left(X_{i}-\mu \right)}^{2}}$	Standard deviation
$X_{rms}$	$\sqrt{\frac{1}{N}\sum_{i=1}^{N}{X_{i}}^{2}}$	Root mean square (RMS)
$X_{sk}$	$\frac{E{\left(X-\mu \right)}^{3}}{{X_{std}}^{3}}$	Skewness (the third standardized moment)
$X_{ku}$	$\frac{E{\left(X-\mu \right)}^{4}}{{X_{std}}^{4}}$	Kurtosis (the fourth standardized moment)
S	$\frac{X_{rms}}{\frac{1}{N}\sum_{i=1}^{N}\left|X_{i}\right|}$	Waveform factor
L	$\frac{X_{peak}}{{(\frac{1}{N}\sum_{i=1}^{N}\sqrt{\left|X_{i}\right|})}^{2}}$	Clearance Factor

**Table 3:** Proportions of microalgae, cyanobacteria, and other SPM given by PFEC.

Site	*Cyanophyta*	*Bacillariophyta*	*Chlorophyta*	Other SPM
CF-1	33.48%	6.08%	8.98%	51.46%
CF-2	27.05%	5.55%	10.39%	57.01%
CF-3	13.46%	11.08%	27.63%	47.83%
CF-4	4.65%	6.69%	34.18%	54.48%
GC-1	38.75%	4.29%	7.93%	49.03%
GC-2	26.96%	8.18%	14.37%	50.49%
GC-3	8.13%	11.70%	25.56%	54.61%
GC-4	5.15%	14.39%	43.10%	37.36%

**Table 4:** Dominant (+++) and common (++) species by microscopy at different sites.

Species	CF-1	CF-2	CF-3	CF-4	GC-1	GC-2	GC-3	GC-4
N8	++	++	+++	+++	++	++	+++	+++
N10				++			++	
N12			++			++		++
N19	++	++	+++	++		++	+++	+++
N20	+++	+++	+++	+++	+++	+++	+++	+++

**Table 5:** Dominant (+++) and common (++) species predicted by PFEC at different sites.

Species	CF-1	CF-2	CF-3	CF-4	GC-1	GC-2	GC-3	GC-4
N8	+++ *	+++ *	+++	+++	++	+++ *	+++	+++
N10				++			++	
N12			++			++		++
N19	++	++	+++	+++ *		+++ *	+++	+++
N20	+++	+++	+++	+++	+++	+++	+++	++ *

* Different from the results in Table 4.

## Data Availability

Dataset available on request from the authors.
